# Determinants of Health-Related Quality of Life in Schizophrenia: Beyond the Medical Model

**DOI:** 10.3389/fpsyt.2018.00712

**Published:** 2018-12-18

**Authors:** Madeline W. Z. Lim, Jimmy Lee

**Affiliations:** ^1^Research Division, Institute of Mental Health, Singapore, Singapore; ^2^Department of Psychosis, Institute of Mental Health, Singapore, Singapore; ^3^Lee Kong Chian School of Medicine, Nanyang Technological University, Singapore, Singapore

**Keywords:** depression, quality of life, schizophrenia, psychoses, outcome, health-related quality of life, determinants

## Abstract

**Background:** Improving Quality of Life (QoL) in Schizophrenia is an important treatment objective in the shift toward person-centered and recovery-oriented care. Health-Related Quality of Life (HRQoL) is a focused aspect of QoL that is directly impacted by healthcare intervention. This aim of the current study was to ascertain the clinical determinants of HRQoL in Schizophrenia and their collective contribution to HRQoL.

**Methods:** 157 stable outpatients with schizophrenia were recruited for this study. Data collected included sociodemographic information and clinical characteristics. HRQoL was assessed on the RAND-36. Psychopathology was assessed on the Positive and Negative Syndrome Scale (PANSS) and functioning measured on the Global Assessment Scale (GAS).

**Findings:** Multiple regression revealed that the Physical Health Component (PHC) of the RAND-36 was associated with positive symptoms (beta = −0.218, *p* = 0.005) and presence of psychiatric comorbidity (beta = −0.215, *p* = 0.003). The Mental Health Component (MHC) was associated with depressive (beta = −0.364, *p* < 0.001) and positive (beta = −0.175, *p* = 0.021,) symptoms. Symptoms, functioning, presence of psychiatric comorbidities, gender and age account for 20.3% of the total variance observed in HRQoL.

**Conclusion:** Depressive and positive symptoms are key clinical determinants of HRQoL in people with schizophrenia. However, the medical model—looking solely at clinical determinants—could not account for a large proportion of variance in HRQoL. Hence, future research beyond the medical model is required to uncover the determinants of HRQoL in Schizophrenia. Identifying these factors will contribute toward developing a holistic and person-centered management plan for people with schizophrenia.

## Introduction

The interest of Quality of Life (QoL) in Psychiatry grew out of the deinstitutionalization of psychiatric patients into the community and the evaluation of their plight was necessary and relevant (1). QoL is broadly defined as “a person's sense of well-being and satisfaction with his/her life circumstances, as well as a person's health status and access to resources and opportunities” (2)^p1225^. Although the illness is heterogeneous, the evaluation of QoL in schizophrenia is even more pertinent when the illness interacts with contextual factors (social and environment factors), even more so than somatic illnesses (3, 4); the illness can be severe, disabling, stigmatizing and chronic, accompanied by cognitive and emotional abnormalities; social, occupational and everyday behavior is affected and treatment involves the side effects of antipsychotic medications (5, 6). Hence, the QoL of psychiatric patients are directly affected by the impact of the illness and the focus on evaluating QoL in this population will highlight this impact on their life situation and their needs (7). In addition, QoL has been shown to be predictive of relapse in schizophrenia (8).

Although QoL seems intuitive (9), there is a lack of clear understanding in its definition and measurement (1). There is a general consensus on the characteristics of QoL as oriented toward the patient's perspective and that it has a subjective and multi-dimensional nature (9). Broadly, it consists of: “a person's (a) satisfaction with his/her life as a whole or *general wellbeing*; (b) observable social and material wellbeing, or *objective QoL*; (c) satisfaction with his/her social and material well-being, or *subjective QoL*; and (d) health and functional status, or *health-related QoL*” (2)^p1226^. Health-Related QoL (HRQoL) has a narrower focus of QoL in medicine (10) directly related to the illness, that refers to the patient's perception of the effect of the illness and its treatment on physical and occupational functioning, psychological state, social interaction, and somatic sensation (6, 11, 12).

With the importance of QoL in schizophrenia, many have studied the determinants of it to enable target areas for intervention (13). Factors explored ranged broadly within sociodemographic, neurocognitive, psychosocial, clinical, and psychopathological domains (14). In the clinical and psychopathological domains, the picture has been unclear and inconclusive as findings of negative and positive symptoms have been mixed (2, 7, 13, 15), in part due to the inconsistencies in usage of QoL measures (7, 14), and although most have found depressive symptoms as the strongest determinant, some have not found so (16–18). In addition, although functioning appears to be an important determinant, it was found that it was not associated with QoL after symptoms were controlled for (19) and that it might be a stronger predictor than symptoms (20). Besides, the determinants of HRQoL, instead of QoL, is worthy of investigation as most researchers have used generic QoL scales to measure general QoL which may be more related to life satisfaction (21).

Although most studies have focused on clinical and/or psychopathological determinants of QoL, recent literature has reported that other factors (2, 22) such as those of psychosocial domains (7, 14, 23, 24) may play a bigger role in QoL. Particularly, Fervaha et al. (22) have brought up that variables beyond the medical model, where they have taken it to be symptoms and even functioning, might have more influential roles.

With the lack of usage of HRQoL measures and consequently the lack of research on clinical determinants of HRQoL in the schizophrenia population, coupled with the inconsistent literature findings on QoL determinants, our study aims to explore the determinants of HRQoL in people with schizophrenia. With considerable evidence that depressive symptoms are the most robust indicator of QoL, we hypothesize that we will be able to replicate this in our study. In addition, against the backdrop of recent findings that factors other than clinical or psychopathological domains may play a bigger role in QoL, we aim to examine the collective impact of symptoms, functioning, and clinical variables (which we term as the medical model) on the HRQoL in people with schizophrenia. In line with these recent findings, we hypothesize that the collective impact of the medical model on HRQoL will be significant but of similar variance as Fervaha et al.'s- 20%.

## Materials and Methods

### Study Participants

One hundred and Fifty-Seven participants were recruited from outpatient clinics at the Institute of Mental Health (IMH) in Singapore. This is the only tertiary psychiatric hospital in the country and provides inpatient, outpatient and community-based services to people with mental illnesses. Eligibility criteria included a clinical diagnosis of schizophrenia, aged between 21 to 69 years old, a duration of illness of more than 1 year and a stable mental state at time of assessment. Participants with neurological conditions, intellectual disability and current substance use were excluded from the study. Signed informed consent was obtained from all the participants and the study was approved by the National Healthcare Group's Domain Specific Review Board.

### Measures

The outcome measure of interest was HRQoL. This was rated using the RAND-36, the most commonly used HRQoL measure (25) that measures general health status related to an individual's ability to function and his or her perceived well-being in physical, mental, and social domains of life. The RAND-36 assesses 8 health concepts: physical functioning, role limitations caused by physical health problems, pain, general health perceptions, emotional well-being, role limitations caused by emotional problems, social functioning, energy/fatigue, and includes a single item that provides an indication of perceived change in health. Two composite scores—Physical Health Composite (PHC) and Mental Health Composite (MHC) can be derived from the scale (25). For the purposes of this study, the norm-based T-scores were not used to derive the 2 composite and 8 subscale scores, as the sociodemographic data were derived from the U.S. Bureau of the Census in 1993. Only the IRT weights (for subscale scores) and the beta weights (for factor-based composite scores) were applied [Refer to Appendix section of Hays and Morales (25)].

Other measures of interest of the current study included clinical characteristics, symptom severity, and functioning. Data on clinical characteristics collected included presence of psychiatric comorbidities, Body Mass Index (BMI), age at first onset of illness, duration of psychiatric illness, duration of psychiatric treatment, and antipsychotic doses in total daily chlorpromazine equivalent (26–28). Symptom severity and functioning were assessed using the Positive and Negative Syndrome Scale (PANSS) (29) and the Global Assessment Scale (GAS) (30), respectively. The PANSS is a comprehensive measure of psychopathology and has been validated in the schizophrenia population in Singapore, comprising of five factors—Positive, Negative, Excitement, Depression, and Cognitive (31).

Socio-demographic information such as age, gender, marital status, and education level and employment status were collected during the interview.

## Statistical Analyses

Statistical analyses were conducted using SPSS 23.0 (SPSS Inc, Chicago, Illinois). Normality tests, association tests and multiple regressions were conducted. The normality of distributions of continuous measures was tested using the Kolmogorov-Smirnov and Shapiro-Wilk test. Bivariate relationships were examined using nonparametric Spearman Rho for continuous variables and nonparametric Kruskal-Wallis test for variables that were not normally distributed. Variables that demonstrated significance were entered into a multiple regression model, using the backward method to derive the most parsimonious model for predictors of each of the composite score—PHC and MHC. A 2-sided *P-*value of less than 0.05 was considered statistically significant. Multicollinearity among predictors was evaluated using tolerance and Variance Inflation Factor (VIF).

## Results

### Sample Characteristics

The sample socio-demographic and clinical characteristics are presented in Table 1 while the descriptive statistics for the clinical assessments and outcome measure—HRQoL are summarized in Table 2.

**Table 1:** Sample characteristics (*n* = 157).

**Characteristics**	**Mean (SD) or no. (%)**
**GENDER**	
Male	88 (56.1)
Female	69 (43.9)
**MARITAL STATUS**	
Never married	123 (78.3)
Married	15 (9.6)
Separated	2 (1.3)
Divorced	15 (9.6)
Widowed	2 (1.3)
**EMPLOYMENT STATUS**	
Employed	75 (47.8)
Unemployed	82 (52.2)
**EDUCATION LEVEL**	
Primary/lower	29 (18.5)
Secondary	40 (25.5)
Post-secondary	88 (56.1)
**PRESENCE OF PSYCHIATRIC COMORBIDITIES**	
No	38 (24.2)
Yes	119 (75.8)
Body Mass Index, kg/m^2^	26.2 (4.8)
Age, years	39.9 (9.5)
Age of first onset of illness, years	24.0 (6.7)
Duration of psychiatric illness, years	16.2 (8.7)
Duration of psychiatric treatment, years	15.5 (8.6)
Antipsychotic dose, mg	490.3 (418.3)

*Antipsychotic dose is in total CPZ equivalent*.

**Table 2:** PANSS, GAS, and RAND-36 score.

**Ratings**	**Mean (SD)**
**PANSS**	
Positive	7.3 (4.1)
Negative	7.8 (3.2)
Excitement	4.2 (1.7)
Depression	5.6 (2.4)
Cognitive	4.3 (1.8)
GAS	62.1 (11.3)
RAND-36 weighted/factored scores physical functioning	441.8 (91.9)
Role Limitations due to physical health problems	224.1 (71.1)
Pain	117.8 (42.5)
General health perceptions	261.3 (77.5)
Emotional well-being	214.5 (69.7)
Role limitations due to emotional problems	154.8 (47.2)
Social functioning	137.0 (38.9)
Energy/fatigue	170.6 (68.2)
Physical health composite	307.0 (62.4)
Mental health composite	214.2 (57.6)

*PANSS, Positive and Negative Syndrome Scale; GAS, Global Assessment Scale*.

### Bivariate Relationships With PHC and MHC

Bivariate relationships between the outcome measure (HRQoL) and sociodemographic factors, clinical characteristics, and clinical assessments are presented in Table 3. Presence of psychiatric comorbidities was associated with lower PHC scores (χ^2^ = 9.043, *df* = 1, *p* = 0.003). Better functioning was associated with higher PHC scores (ρ = 0.270, *p* = 0.001). Higher PANSS Positive (ρ = −0.251, *p* = 0.001), Excitement (ρ = −0.198, *p* = 0.013), Depression (ρ = −0.232, *p* = 0.003) and Cognitive (ρ = −0.170, *p* = 0.033) factor scores were significantly associated with lower PHC scores.

**Table 3:** Bivariate relationships with RAND-36 physical health composite and mental health composite.

	**PHC median**	**PHC Statistic**	***p*-value**	**MHC median**	**MHC Statistic**	***p*-value**
Gender	–	1.249	0.264	–	4.777	**0.029**
Male	302.71	–	–	197.82	–	–
Female	326.41	–	–	215.23	–	–
Education level	–	3.392	0.183	–	1.382	0.501
Marital status	–	7.106	0.130	–	2.52	0.641
Employment status	–	0.022	0.883	–	0.046	0.83
Presence of psychiatric comorbidities	–	9.043	**0.003**	–	5.432	**0.02**
No	327.25	–	–	211.25	–	–
Yes	288.24	–	–	194.91	–	–
Body Mass Index, kg/m^2^	–	0.013	0.87	–	0.122	0.127
Age in years	–	−0.031	0.697	–	0.139	0.082
Age of first onset of illness, years	–	0.068	0.395	–	0.150	0.062
Duration of psychiatric illness, years	–	−0.086	0.283	–	0.032	0.693
Duration of psychiatric treatment, years	–	−0.087	0.28	–	0.048	0.548
Antipsychotic dose, mg	–	−0.087	0.283	–	−0.065	0.421
GAS	–	0.270	**0.001**	–	0.317	** < 0.001**
PANSS positive	–	−0.251	**0.001**	–	−0.293	** < 0.001**
PANSS negative	–	−0.064	0.427	–	−0.079	0.323
PANSS excitement	–	−0.198	**0.013**	–	−0.105	0.19
PANSS depression	–	−0.232	**0.003**	–	−0.391	** < 0.001**
PANSS cognitive	–	−0.170	**0.033**	–	−0.059	0.463

*In bold are correlation or independent tests significant at p < 0.05 level*.

*PHC, Physical Health Composite; MHC, Mental Health Composite; PANSS, Positive and Negative Syndrome Scale; GAS, General Assessment Scale*.

Males had lower MHC scores (χ^2^ = 4.777, *df* = 1, *p* = 0.029). Presence of psychiatric comorbidities was associated with lower MHC scores (χ^2^ = 5.432, *df* = 1, *p* = 0.02). Better functioning (ρ = 0.317, *p* < 0.001) was associated with higher MHC scores. Higher PANSS Positive (ρ = −0.293, *p* < 0.001) and Depression (ρ = −0.391, *p* < 0.001) factor scores were associated with lower MHC scores.

### Multiple Regression Modeling

In the multivariate models, PANSS Positive Factor and the presence of psychiatric comorbidities were found to be significant predictors of PHC (adjusted *R*^2^ = 8.7%). PANSS Depression Factor and PANSS Positive Factor were found to be significant predictors of MHC (adjusted *R*^2^ = 18.8%) with PANSS Depression Factor as the stronger predictor (Table 4).

**Table 4:** Multiple regression models.

	**Standardized *B* coefficient**	***t***	***p*-value**	***F* change**	**Sig. *F* change**
**PHC MODEL**					
Presence of psychiatric comorbidities	−0.215	−2.807	0.006	7.878	0.006
PANSS positive	−0.218	−2.842	0.005	8.666	0.004
**MHC MODEL**					
PANSS depression	−0.364	−4.834	< 0.001	31.856	0.000
PANSS positive	−0.175	−2.327	0.021	5.415	0.021

*See Table 2 for abbreviations*.

*PHC Model: Adjusted R^2^ = 0.087*.

*MHC Model: Adjusted R^2^ = 0.188*.

### Bivariate Correlation

Bivariate relationships between the scale items of HRQoL and PANSS Positive and Depression Factors are presented in Table 5. Higher PANSS Positive Factor score was associated with lower scores on some of the scale items of the PHC: largely on role limitations due to physical health problems and pain, and moderately on general health perceptions. Higher PANSS Positive Factor score was associated with lower scores on all scale items of the MHC. Higher Depression Factor score was also associated with lower scores on all scale items of the MHC.

**Table 5:** Spearman correlation between HRQoL scale items and PANSS positive and depression factor.

**HRQoL domain**	**PANSS positive**	**PANSS depression**
**PHYSICAL HEALTH COMPONENT**		
Physical functioning	**rs = −0.086**, *p* = 0.284	–
Role limitations due to physical health problems	**rs = −0.277**, *p* = 0.000	–
Pain	**rs = −0.230**, *p* = 0.004	–
General health perceptions	**rs = −0.173**, *p* = 0.031	–
**MENTAL HEALTH COMPONENT**		
Emotion well-being	**rs =** –**0.206**, *p* = 0.010	**rs = −0.382**, *p* = 0.000
Role limitations due to emotional problems	**rs = −0.259**, *p* = 0.001	**rs = −0.239**, *p* = 0.003
Social functioning	**rs = −0.290**, *p* = 0.000	**rs = −0.303**, *p* = 0.000
Energy/fatigue	**rs = −0.232**, *p* = 0.003	**rs = −0.327**, *p* = 0.000

*In bold are correlation significant at the p < 0.05 level; or p < 0.01 level*.

### Multiple Regression Modeling-Medical Model

The collective variance explained by the medical model (PANSS symptoms, GAS, presence of psychiatric comorbidities) with gender and age entered as covariates is 9.1% for PHC and 20.3% for MHC.

## Discussion

This study intended to determine the clinical determinants of HRQoL in a sample of outpatients with schizophrenia. The predictors of the MHC of HRQoL were depressive and positive symptoms, and positive symptoms and psychiatric comorbidities for the physical health component (PHC).

### Depressive Symptoms

Our finding of depressive symptoms as a key determinant of HRQoL in people with schizophrenia is consistent with many other QoL studies (12, 14, 32, 33). Even though the effects of depressive symptoms was only seen on the MHC, it is still consistent with the literature as Meijer et al. (34) had found that the largest proportion of effect on general quality of life was mediated by the mental health component summary scale of the HRQoL- SF36 measure in their study. In addition, the RAND-36 comprised of two components (PHC and MHC) have equal scale items (4 each). Other QoL measures, such as the World Health Organization Quality of Life Measure-Brief Version (WHOQOL-BREF) and schizophrenia-specific HRQoL (S-QoL) tend to under-represent physical health domains; only one out of four domains for WHOQOL-BREF and one out of eight domains for S-QoL take into consideration the physical health effects on QoL. This also illustrates the lack of emphasis on physical health when evaluating one's QoL. The effects of depressive symptoms on MHC persist even after adjusting for the level of functioning. Outpatients with schizophrenia with higher severity of depressive symptoms were found to have lower HRQoL scores—particularly MHC. Compared to positive symptoms, depressive symptoms had a greater effect on MHC.

There have been critiques that the relationship between depressive symptoms and QoL might be due to one's mood affecting one's subjective QoL rating, in which patients with depressive symptoms would rate their QoL lower (35). However, the meta-analysis results from Eack and Newhill (2) and the longitudinal study from Cohen et al. (15) suggested otherwise as the former found that it was general psychopathology (depressive and anxiety symptoms) that was most strongly related to both objective and subjective indicators (not only subjective) of QoL while the latter found that depression scores were predictors of baseline QoL score as well as follow-up QoL score at 18th month. Hence, depressive symptoms do not invalidate QoL assessment but that it will affect QoL (36).

The exact way that depression symptoms affect QoL or HRQoL is not well established and studied, although it has been suggested that it results in reduced self-reported life satisfaction and increased social isolation as it decreases interactions with friends and peers (37). Cohen et al.'s (15) longitudinal study of QoL in older adults (≥55 years old) have also suggested that the relationship between QoL and depressive symptoms are bidirectional compared to other clinical symptoms. Further studies on the relationship between depression symptoms and QoL is warranted.

### Positive Symptoms

Findings on the association of positive symptoms on QoL have been varied (2, 13) where some have found associations (15, 22, 23, 33, 38, 39) while most have not (12, 14, 32, 40–47). In our study where HRQoL was employed, we found positive symptoms to be a significant predictor of both PHC and MHC. Eack and Newhill's (2) meta-analysis suggested that this disparate finding might be due to positive symptoms having strongest relationship with HRQoL, smaller associations with subjective QoL and general well-being, and no relationship with objective QoL. Hence, one of the reasons most studies did not find significant results for positive symptoms might be due to the different QoL measures used.

In our study, positive symptoms were significantly associated with all aspects of MHC and most aspects of PHC (except physical functioning). Although depressive symptoms had a larger role to play in MHC than positive symptoms, positive symptoms (a mental health problem) can affect physical health domains of pain, further adding on to role limitations due to physical health problems, and affect general health perception. This underscores the significance of positive symptoms and thus, it should remain a treatment focus.

### Functioning

Contrary to our expectations, we did not find functioning to be a significant predictor of HRQoL. This could be attributed to the measure that was used. In the GAS, functioning is conflated with severity of symptoms as the consideration of whichever was more severe-(1) symptoms or (2) social, occupational, or psychological functioning was used for the final rating score. Another reason could be that we did not account for the confounding variable- current or chronic medical comorbidities. Schizophrenia or severe mental illnesses often co-occurs with chronic medical conditions especially cardiovascular diseases and diabetes which consequently results in early mortality (48–50). In one study, a greater number of medical conditions was found to be associated with greater depressive symptoms (51) even after schizophrenia symptoms were controlled for. In another study, chronic medical conditions were found to affect QoL via physical impairment (increased deficits in physical body function and activity) directly and indirectly via reduced positive affect (52). Hence, accounting for the presence of current medical comorbidities or chronic comorbidities could alter our results as it might have an inverse correlation with functioning and a positive correlation with depressive symptoms.

### Negative Symptoms

Negative symptoms was not a significant predictor of HRQoL in our study, contrary to some studies (12, 17, 18, 38, 40, 42, 47, 53). It has been argued that the reason subjective QoL scales are not sensitive to negative symptoms might have been due to the way negative symptoms have been assessed (54). Savill et al. (54) suggested that older scales depend on behavioral referents (expressive deficits) and using a newer scale that assesses both experiential and expressive symptoms, they found that SQoL was significantly associated with experiential but not expressive type of negative symptoms. In addition, it has been found that subjective QoL measures is predicted by depressive symptoms and objective QoL measures is predicted by negative symptoms (2, 38, 43–45). Thus, this could also explain why we did not find negative symptoms to be a significant predictor in our findings when using a subjective HRQoL measure.

With the differing results between objective and subjective QoL measures, some might be skeptical about the reliability and validity of self-rated instruments in people with schizophrenia due to the cognitive deficits (9, 55) and lack of insight (56). While objective data of functioning (e.g., how many hours do you sleep?) is important, QoL captures the global evaluation of functioning (e.g., how well do you sleep?) and the highly personalized evaluation of functioning (e.g., how satisfied are you with your sleep?) (9). Furthermore, Narvaez et al. (45) found that apart from the low correlation between subjective QoL and objective QoL, their relationships with patient characteristics varied which suggests that they may be separable and possibly complementary constructs. Hence, both types of QoL data are essential.

### Medical Model

When symptoms, functioning and presence of psychiatric comorbidities, including age and gender entered as covariates, were added in the model, the variance explained for PHC was 9.1 and 20.3% for MHC. This is in agreement with several reports (2, 14, 22) that have found small variances explained by symptoms, clinical factors and functioning. It has been proposed that psychosocial influences (2) or patient characteristics have more crucial or mediational role (57) in explaining HRQoL (4) and several recent studies have shown support for this (7, 24, 58–62).

### Limitations and Future Directions

There are several limitations and weaknesses in our study. This is a cross-sectional study in which causal relationships between the variables cannot be determined. Future studies might consider alternate designs to examine how the relationship between clinical predictors and HRQoL changes throughout the course of schizophrenia. Our study sample consists of stable outpatients with chronic schizophrenia and hence, our results are not generalizable to inpatients or individuals with acute or more severe conditions. Future studies should consider comparing the HRQoL of both inpatients and outpatients as it has been suggested that there is stronger relationships between clinical symptoms and QoL for outpatients since the symptoms would be more disabling for them to survive and progress in the community (2). The RAND-36 used in this study is not a disease-specific health-related QoL measure, which can limit the identification of significant factors specific to people with schizophrenia. However, our results are congruent with the main findings of similar studies, especially those that have used disease-specific HRQoL measures (14, 32). Depressive symptoms in this study were not assessed on a depression specific scale. The PANSS Depression factor and PANSS Negative factor, though validated in our population, may not be a comprehensive evaluation of depressive symptoms and negative symptoms respectively. Nevertheless, for PANSS Depression factor, we were able to reproduce previous findings that depression was the predominant clinical factor. The PANSS Negative factor does not evaluate experiential symptoms and as discussed above, it may be one of the reasons for the lack of association with HRQoL. Future research should utilize one that comprehensively evaluates negative symptoms. There is a lack of a global composite score for the RAND-36; with just the MHC, it was difficult to conclude that the results relate to HRQoL as a whole.

A meta-analysis revealed that neurocognitive deficits has a significant small-moderate relationship with objective QoL measures (functional status rated by either client or clinician) but insignificant relationship with subjective QoL (client satisfaction) (63) whereas, three recent studies suggest that cognitive deficits when assessed from the patients' point of view, produces significant relationship with QoL (64–66). This warrants the need to further investigate the role cognition plays in QoL of patients with schizophrenia. As mentioned above, functioning assessed on the GAS included symptoms severity in the final score. A pure functioning scale would have been more appropriate and is recommended for future studies. Furthermore, we did not account for the confounding effects of current or chronic medical comorbidities and level of insight (35) as well as the role of the side effect of medications (1, 10). Social cognitive impairments may also play a more important role than cognition in the QoL of people with schizophrenia (64, 67). This underscores the need to include such variables in the model for future studies.

Our consideration of the relationships amongst the predictors might be too simplistic as it has been postulated that the relationships might not be straightforward (68). Future studies should consider the complex relationships amongst the predictors when designing a study and the usage of more complex statistical analysis such as structured equation modeling to analyze such data. In addition, QoL might have a dual role of affecting clinical symptoms and being an outcome in itself (15), hence using QoL as a mediator and as an outcome should also be considered (10).

Although HRQoL helps us to lay aside the aspects of QoL that may not be affected by healthcare interventions, its weakness is its focus on the deficits of functioning and not including positive functioning i.e., positive emotions and psychological resources (4). Hence, future studies should consider the inclusion of well-being scales. Future studies should also consider the use of individualized QoL instruments as QoL measures have not been developed by service-users but is assumed that all individuals attach the same relative importance or priority to different aspects of life (69). However, this may not necessarily be so (70) and thus, there might not be one standard way to evaluate.

## Conclusion

This study affirms depressive symptoms as the most important clinical factor that affects the health-related quality of life in people with schizophrenia. Positive symptoms and presence of psychiatric comorbidities have significant but weaker effects on HRQoL. Our results suggest that we should pay attention to depressive symptoms and positive symptoms in order to improve the HRQoL of individuals with schizophrenia. Our results also suggest that beyond the medical model, there are other factors or variables that play crucial roles in determining a patient's level of HRQoL. Identifying these factors will contribute toward developing a holistic and person-centered management plan for people with schizophrenia.

## Ethics Statement

This study was carried out in accordance with the recommendations of the National Healthcare Group's Domain Specific Review Board (Singapore), with written informed consent from all participants. All participants gave written informed consent in accordance to the Declaration of Helsinki. The study protocol was approved by the National Healthcare Group's Domain Specific Review Board (Singapore).

## Author Contributions

ML managed the literature searches and statistical analyses, and wrote the first draft of the manuscript. JL interpreted the data and contributed in writing the manuscript. Both authors contributed to the revisions of the manuscript, and have read and approved the final manuscript.

### Conflict of Interest Statement

The authors declare that the research was conducted in the absence of any commercial or financial relationships that could be construed as a potential conflict of interest.
